# Digital assessment of cognition in neurodegenerative disease: a data driven approach leveraging artificial intelligence

**DOI:** 10.3389/fpsyg.2024.1415629

**Published:** 2024-07-05

**Authors:** David J. Libon, Rod Swenson, Catherine C. Price, Melissa Lamar, Stephanie Cosentino, Ondrej Bezdicek, Mitchel A. Kling, Sean Tobyne, Ali Jannati, Russell Banks, Alvaro Pascual-Leone

**Affiliations:** ^1^Department of Geriatrics and Gerontology, New Jersey Institute for Successful Aging, Rowan-Virtua School of Osteopathic Medicine, Glassboro, NJ, United States; ^2^Department of Psychology, Rowan University, Glassboro, NJ, United States; ^3^Department of Psychiatry and Behavioral Health, University of North Dakota School of Medicine and Health Sciences, Grand Forks, ND, United States; ^4^Department of Clinical and Health Psychology, University of Florida, Gainesville, FL, United States; ^5^Rush Alzheimer's Disease Center and the Department of Psychiatry and Behavioral Sciences, Rush University Medical Center, Chicago, IL, United States; ^6^Columbia University Medical Center, Department of Neurology, Cognitive Neuroscience Division, Taub Institute and Sergievsky Center, New York, NY, United States; ^7^Department of Neurology and Center of Clinical Neuroscience, First Faculty of Medicine, Charles University, Prague, Czechia; ^8^Linus Health, Boston, MA, United States; ^9^Department of Neurology, Harvard Medical School, Boston, MA, United States; ^10^Hinda and Arthur Marcus Institute for Aging Research and Deanna, Sidney Wolk Center for Memory Health, Hebrew Senior Life, Boston, MA, United States

**Keywords:** digital assessment of cognition, executive control, episodic memory, Alzheimer's disease, mild cognitive impairment, Boston Process Approach, Philadelphia (repeatable) Verbal Learning Test, Backward Digit Span Test

## Abstract

**Introduction:**

A rapid and reliable neuropsychological protocol is essential for the efficient assessment of neurocognitive constructs related to emergent neurodegenerative diseases. We developed an AI-assisted, digitally administered/scored neuropsychological protocol that can be remotely administered in ~10 min. This protocol assesses the requisite neurocognitive constructs associated with emergent neurodegenerative illnesses.

**Methods:**

The protocol was administered to 77 ambulatory care/memory clinic patients (56.40% women; 88.50% Caucasian). The protocol includes a 6-word version of the Philadelphia (repeatable) Verbal Learning Test [P(r)VLT], three trials of 5 digits backward from the Backwards Digit Span Test (BDST), and the “animal” fluency test. The protocol provides a comprehensive set of traditional “core” measures that are typically obtained through paper-and-pencil tests (i.e., serial list learning, immediate and delayed free recall, recognition hits, percent correct serial order backward digit span, and “animal” fluency output). Additionally, the protocol includes variables that quantify errors and detail the processes used in administering the tests. It also features two separate, norm-referenced summary scores specifically designed to measure executive control and memory.

**Results:**

Using four core measures, we used cluster analysis to classify participants into four groups: cognitively unimpaired (CU; *n* = 23), amnestic mild cognitive impairment (MCI; *n* = 17), dysexecutive MCI (*n* = 23), and dementia (*n* = 14). Subsequent analyses of error and process variables operationally defined key features of amnesia (i.e., rapid forgetting, extra-list intrusions, profligate responding to recognition foils); key features underlying reduced executive abilities (i.e., BDST items and dysexecutive errors); and the strength of the semantic association between successive responses on the “animal” fluency test. Executive and memory index scores effectively distinguished between all four groups. There was over 90% agreement between how cluster analysis of digitally obtained measures classified patients compared to classification using a traditional comprehensive neuropsychological protocol. The correlations between digitally obtained outcome variables and analogous paper/pencil measures were robust.

**Discussion:**

The digitally administered protocol demonstrated a capacity to identify patterns of impaired performance and classification similar to those observed with standard paper/pencil neuropsychological tests. The inclusion of both core measures and detailed error/process variables suggests that this protocol can detect subtle, nuanced signs of early emergent neurodegenerative illness efficiently and comprehensively.

## Introduction

Alzheimer's disease (AD) represents one of the most intractable, malignant, and widespread public health problems. Recent data suggest that in the United States, ~6.5 million people are affected by dementia due to AD. When prodromal neurological conditions associated with AD, such as mild cognitive impairment (MCI), are considered, 17% of Americans could be affected (Alzheimer's Association, 2023). In 2023, Medicare/Medicaid spent over 200 million dollars financing the care of people with AD (Alzheimer's Association, 2023). Importantly, recent pharmacological advances now offer the possibility for some relief and disease modification for those diagnosed with MCI and mild dementia due to AD (Sims et al., 2023; van Dyck et al., 2023).

A key element in this rapidly developing landscape revolves around periodic and timely assessments for signs and symptoms of AD and related dementias (ADRD), including MCI. In this context, attention has recently been focused on how services provided by primary care providers (PCPs) can be leveraged to detect and track cognitive symptoms and potentially identify candidates for currently available and emerging disease-modifying treatments for AD. Bradford et al. (2009) noted that the Institute of Medicine (2001) has defined timely diagnosis and care for people with dementia, such as AD, as a primary aim to improve healthcare for Americans. However, Bradford et al. (2009) also enumerated a variety of barriers that prevent effective screening, including insufficient knowledge among PCPs regarding the selection, administration, and interpretation of tests used to screen for ADRD. Mattke et al. (2023) emphasized these comments and called not only for new, brief cognitive assessment tools that can be reasonably deployed and easily integrated into clinical workflow given the limited amount of time PCPs have with their patients but also for a re-examination and update of current insurance reimbursement policies for these much-needed services.

The recent amalgam of neuropsychological tests administered and scored using digital technology could be the means to overcome these challenges (see Libon et al., 2022, for a review). Libon et al. (2022, 2023a) have called attention to a number of important systematic benefits associated with the deployment of digital assessment technology. These benefits include excellent reliability, the absence of subjectivity with respect to test administration and scoring, and the ease with which non-physician personnel can be trained in the administration of digital tests. The most important benefit is probably the power of this technology to unmask, measure, and define key neurocognitive constructs well-known to be associated with ADRD and MCI.

A prime example of the effectiveness of this new technology is the combination of the classic paper and pencil clock drawing test (Libon et al., 1993, 1996; Cosentino et al., 2004) with digital assessment technology to create the digital clock drawing test (Penney et al., 2011a,b,c; Libon et al., 2014, 2022, 2023a; Souillard-Mandar et al., 2016, 2021). For example, recent research using the digital clock drawing test has uncovered a variety of occult or hidden decision-making latencies and discrete graphomotor behavior as individuals transition from one portion of the test to the next (Libon et al., 2014; 2023a; Piers et al., 2017). Subsequent research has shown that these decision-making or intra-component latencies and other time-based measures can distinguish between memory clinic patients diagnosed with AD vs. vascular dementia (VaD) and between MCI subtypes (Binaco et al., 2020; Davoudi et al., 2021; Matusz et al., 2023). Further, Dion et al. (2020) have recently demonstrated that slower digital clock-drawing intra-component latencies correlate with worse performance on tests that assess various executive abilities. In follow-up research, Dion et al. (2022) found that inaccurately placed numbers within the clock face were negatively associated with semantic, visuospatial, and visuoconstructional operations, as well as reduced connectivity from the basal nucleus of Meynert to the anterior cingulate cortex. In additional research, Emrani et al. (2021a,b); and Emrani et al. (2023) administered the Backward Digit Span and other tests to memory clinic patients with suspected MCI, where all groups scored 100% correct using commonly used paper and pencil analog metrics. Interestingly, as observed with the digital clock drawing derived research metrics described above, an analysis of latency or reaction time to generate each digit backward was able to differentiate between MCI vs. non-MCI subgroups.

In addition to the latency and graphomotor behavior described above, there is now much interest regarding how acoustic/prosodic behavior can be extracted while patients are administered neuropsychological tests (see Geraudie et al., 2021; Mahon and Lachman, 2022, for a review). Libon et al. (2023b) found that research participants classified with amnestic MCI or dysexecutive MCI did not differ with respect to the number of responses generated on the “animal” fluency test. Nonetheless, the creation of an index that assessed acoustic parameters that included pitch, decibel, jitter, shimmer, and other acoustic behaviors resulted in significant between-group differences.

All of this research suggests that digital assessment technology is parsimonious with respect to the time necessary for test administration and scoring and is able to unmask, measure, and define key neurocognitive constructs commonly understood to be associated with ADRD and MCI but is often difficult to measure with paper/pencil testing. A notable advantage of digital assessments is their capacity to automatically score the errors made on neuropsychological tests. This feature provides valuable insights into the strategies or processes patients use during test completion, offering a deeper understanding of their cognitive functioning.

This approach to neuropsychological assessment is known as the Boston Process Approach, a method of neuropsychological inquiry pioneered and championed by Kaplan (1988, 1990); (see Geschwind and Kaplan, 1962; Lamar et al., 2010; Libon et al., 2018). In the current research, ambulatory care and memory clinic research participants were assessed with a 10-min digitally administered and scored neuropsychological protocol measuring executive, language-related, and episodic memory abilities.

The goals of the current research were to assess (1) how well a brief, digitally administered, remote-capable neuropsychological protocol can classify participants into meaningful clinical groups; (2) explore how this technology is able to tally errors and define the processes used by participants during testing; and (3) conduct a preliminary comparison between this remote-capable, digitally administered protocol vs. a comprehensive traditional paper and pencil neuropsychological assessment.

## Methods

### Participants

Participants in the current research (*n* = 77; 56.40% women; 88.50% Caucasian) came from three sources, including the Rowan University, New Jersey Institute for Successful Aging, Memory Assessment Program (MAP; *n* = 35); outpatient referrals for neuropsychological assessment for suspected dementia (*n* = 12); and the Rowan University Departments of Geriatrics and Family Medicine outpatient ambulatory care services (*n* = 30). The NJISA MAP program provides a comprehensive outpatient evaluation and work-up for suspected alterations involving cognition and personality/behavior. MAP patients were scheduled for three outpatient visits involving the administration of a neuropsychological protocol, an evaluation by a board-certified geriatric psychiatrist, and an evaluation by a clinical worker. An MRI study of the brain using a 3-T magnet and serum tests, including a CBC, CMP, thyroid/B12, and an analysis of lipids, was obtained. Participants referred by their primary care provider for outpatient neuropsychological assessment because of suspected dementia (*n* = 12) underwent the same neuropsychological evaluation as MAP patients.

Participants from the Rowan Departments of Geriatrics and Family Medicine were not referred or assessed clinically and did not undergo outpatient neuropsychological assessment. These participants were recruited for ongoing research on the development of digital neuropsychological assessment technology. Participants were excluded from this study if English was not their first language or if there was any history of head injury, substance abuse, a major psychiatric disorder such as major depression, another neurologic illness such as epilepsy, or metabolic disorders such as B12, folate, or a thyroid deficiency. For MAP and outpatient participants referred for neuropsychological evaluation, a knowledgeable family member was available to provide information regarding functional status. This study was approved by the Rowan University Institutional Review Board, and consent was obtained that was consistent with the Declaration of Helsinki.

### Neuropsychological evaluation

The paper and pencil neuropsychological protocol used for clinical evaluation (*n* = 47) assessed six domains of cognitive abilities, including executive control/working memory, graphomotor information processing speed, general intellectual abilities, language/lexical access, visuospatial/visuoconstructional abilities, and episodic memory. These neuropsychological tests were expressed as *z*-scores derived from available normative data or demographically corrected scores provided by Heaton et al. (2004). A partial list of these tests can be found in Supplementary Table 1.

### The digital assessment of cognition

The digital neuropsychological protocol used in the current research consisted of three tests. The order of test administration was as follows: Two 6-word Philadelphia (repeatable) Verbal Learning Tests [P(r)VLT] – immediate free recall test trials; the semantic/”animal” fluency test (60 s); three trials of 5-digits backward from the Backwards Digit Span Test (BDST); a 6-item depression/anxiety screening inventory; and concluding with the P(r)VLT-delay free recall and delay recognition test conditions. Throughout the test administration, participants spoke their responses out loud, and the iPad recorded all of their speech for later processing and analysis. The protocol was administered using an 11-inch Apple iPad Pro. A trained examiner proctored the test; however, all test instructions were delivered verbally by the iPad. During the assessment, the iPad was kept in portrait orientation while lying on a flat surface.

### Core digital outcome measures

#### Verbal episodic memory [P(r)VLT]

This neurocognitive domain was assessed with a 6-word version of the Philadelphia (repeatable) Verbal Learning Test [P(r)VLT]. The P(r)VLT was modeled after the California Verbal Learning Test (CVLT, Delis et al., 1987). The 6-word version of the P(r)VLT used in the current research was constructed from the original 9-word version described by Price et al. (2009). For this test, two words were drawn from three semantic categories (fruits, tools, and school supplies). Each word was spoken by the iPad one second at a time. Two immediate free recall test trials were administered (range 0–6). After each free recall test trial, the iPad asked the participant to verbally recall as many words as possible. After a delay, a free recall was assessed (range 0–6). For the delayed recognition test condition, participants saw and heard the iPad read groups of three words. Each group of three words contained one of the original target items, one prototypic semantic foil (e.g., apple, hammer), and one generic semantic foil (e.g., peaches, wrench). Participants were asked to touch one word in the original word list (Table 1).

**Table 1:** Digital neuropsychological protocol: outcome variables.

	**Core outcome variables**	**Error and process outcome variables**
P(r)VLT	Free recall: immediate and delay free recall for words recalled (Price et al., 2009) Recognition test performance: correct hits (Price et al., 2009)	Free recall: semantic cluster responses; extra-list intrusion errors; perseverations (Price et al., 2009) Contrast comparisons: immediate free recall, trial 2 vs. delay free recall & delay recognition; delay free recall vs. delay recognition (Price et al., 2009) Recognition foils: total recognition foils; prototypic false positive foils; generic false positive foils (Price et al., 2009) Recognition latency: latency/reaction time for correct recognition trials Recognition correct hit/recognition false positive ratio: ([correct recognition hits/correct recognition hits + total recognition foils)]; (see Rascovsky et al., 2007)
BDST	Aggregate performance: percent ANY order recall; percent SERIAL order recall (Lamar et al., 2007, 2008)	Transpositions errors: total out-of-sequence errors (Emrani et al., 2018) Dysexecutive errors: sum of within-trial capture errors, between-trial capture errors; and perseveration (Emrani et al., 2018)
“Animal” fluency	Total correct responses (Giovannetti et al., 1997)	“Animal” Association Index (AI; Giovannetti et al., 1997)

P(r)VLT, Philadelphia (repeatable) Verbal Learning Test; BDST, Backward Digit Span Test.

#### Verbal working memory (BDST)

Three trials of five numbers backward were administered. These test trials were drawn from the Backward Digit Span Test (BDST; see Lamar et al., 2007, 2008) and Emrani et al. (2021a,b); Emrani et al. (2023). The original BDST of Lamar et al. (2007, 2008) consisted of seven trials of 3-, 4-, and 5-digit span lengths for a total of 21 trials. As originally constructed, all 4- and 5-span test trials contained contiguous numbers that were placed in strategic positions. For example, in 5-span test trials, contiguous numbers were placed in the middle three-digit positions (e.g., 16,579). In the current research, the BDST was administered using standard Wechsler Digit Span Backward procedures with the exception that the discontinuation rule was not applied, i.e., all participants were assessed with all three test trials, and the iPad recorded all responses.

Performance on this test was expressed as a percentage of serial order and a percentage of any order recalls, as described by Lamar et al. (2007, 2008). Percent SERIAL order recall tallied the total number of digits correctly recalled in accurate serial position divided by the total possible correct responses (i.e., three trials of 5 digits = 15 total responses). As described by Lamar et al. (2007, 2008), this variable was created to assess verbal working memory. Percent ANY order recall is the sum total of every digit correctly recalled regardless of serial order position, divided by the total possible correct responses (i.e., 15 total responses). This variable was created to assess less complex aspects of working memory, characterized mainly by short-term or immediate storage and rehearsal mechanisms.

#### Lexical access/semantic fluency

This neurocognitive domain was assessed with the semantic, “animal” verbal fluency test (Giovannetti et al., 1997), where participants were given 60 s to verbally generate animal exemplars. The number of correct responses was tallied, and all responses were recorded by the iPad.

#### Digital core summary scores

Standardized summary scores (*z*-scores) designed to express the severity of episodic memory and dysexecutive impairment were compiled. The memory index score (MIS) averaged P(r)VLT total free recall and recognition hits (Delis et al., 1987; Libon et al., 2011). The executive index score (EIS) averaged total output on the “animal” fluency test and BDST percent SERIAL order recall, as described by Eppig et al. (2012). The left-hand column in Table 1 lists all the core summary scores.

### Digital error and process outcome measures

#### P(r)VLT recall and retention

This behavior was assessed by comparing (1) immediate free recall, trial 2 vs. delay free recall, (2) immediate free recall, trial 2 vs. recognition hits, and (3) delayed free recall vs. recognition hits, as listed in Table 1.

#### P(r)VLT free recall semantic cluster responses, extra-list intrusion errors, perseverations, and recognition foils

Free recall semantic cluster responses, extra-list intrusion errors, perseverations, and recognition foils were scored following protocols from prior research (Delis et al., 1987; Price et al., 2009; Libon et al., 2011). All immediate and delayed free recall cluster responses, extra-list intrusion errors, perseverations, and recognition foils were tallied to create single scores, respectively.

#### P(r)VLT recognition test performance

The mean latency or reaction time (msecs) for all correct recognition test items (range 0–6) was tallied. The relation between correctly identifying and rejecting recognition hits was assessed with a recognition hit/recognition false positive ratio: [recognition hits/recognition hits + total incorrect recognition foils]. This formula (range 0.00–1.00) is modeled after Rascovsky et al. (2007). A higher score suggests greater numbers of correctly identified recognition in relation to fewer numbers of incorrectly identified recognition foils.

#### BDST total transposition errors

The total number of out-of-sequence or transposition errors was tallied as described by Hurlstone et al. (2014) and Emrani et al. (2018).

#### BDST dysexecutive errors

A variety of errors were coded, including perseverations when patients repeated a digit within a given trial (i.e., 16579–“97569”); within-trial capture errors when participants grouped contiguous numbers in serial order (i.e., 16579–“95671” or “97651”); and between trial capture errors when participants incorporated a digit or digits from either the immediately preceding test trial or two prior test trials to create contiguous numbers in a serial order. All three errors were summed to create a single BDST item error score.

#### The “animal” Fluency Association Index

All animal exemplars were coded (yes = 1, no = 0) on six attributes: size (big, small); geographic location (foreign, local); habitat (farm, pet, water, prairie, forest, African-jungle, Australian, widespread); zoological class (insects, mammals, birds, fish, amphibians, and reptiles); zoological orders, families, and related groupings (feline, cervidae, and rodenta); and diet (herbivore, carnivore, and omnivore). The “animal” fluency AI is the cumulative number of shared attributes between all successive responses divided by the total number of words generated minus one. The sum of the shared attributes was divided by the number of responses minus one to guard against inflating the AI, as attributes from the first response are never actually figured into the sum of the scaled attributes. This index was devised to measure the strength of the semantic association between consecutive responses. The right-hand column in Table 1 lists and describes all digital error and process outcome measures.

### Statistical analysis

All analyses were calculated using SPSS (v29). A *k*-means cluster analysis using four of the six core L-DNP outcome measures was used to classify participants into their respective groups. These variables included P(r)VLT delayed free recall, P(r)VLT recognition hits, BDST serial order recall, and total “animal” fluency responses. This decision was made to mirror prior research (Libon et al., 2009, 2014), where delayed rather than immediate free recall serial list learning test parameters were used to characterize groups. The *k*-means cluster analysis described below specifies four groups. This decision was made based on prior research with memory clinic patients, where groups consistent with amnestic MCI, dysexecutive MCI, dementia, and cognitively unimpaired were studied (Libon et al., 2011; Eppig et al., 2012).

Pearson correlation analyses were undertaken to assess relations between digitally administered core outcome measures and analogous measures from commonly used paper and pencil neuropsychological tests (see Supplementary Table 1). All descriptive and related statistical information for digital core outcome measures is listed and displayed in Supplementary Table 2. As seen in Supplementary Table 2, amnestic MCI and dementia participants generally scored lower on P(r)VLT free recall and recognition core outcome measures compared to other groups. By contrast, dysexecutive MCI participants generally scored lower on core executive measures than other groups.

Following grouping using only the core outcome measures, error and process outcome measures were analyzed using within-group *t*-tests, univariate analysis of variance (ANOVA), and multivariate analysis of variance (MANOVA), as indicated and controlled for age, education, and sex. The Bonferroni correction for multiple comparisons was applied to all analyses.

## Results

### Cluster analysis and demographic information

As displayed in Table 2, the *k*-mean cluster analysis classified participants into groups suggesting cognitively unimpaired neuropsychological test performance (CU; *n* = 23), amnestic mild cognitive impairment (aMCI; *n* = 17), dysexecutive mild cognitive impairment (dMCI; *n* = 23), and dementia (*n* = 14). Dementia participants were older than CU participants (*p* < 0.004). Moreover, dementia participants had fewer years of education than CU participants (*p* < 0.001). CU vs. dMCI and aMCI vs. dMCI participants did not differ on the MMSE. However, CU participants outperformed other groups on the MMSE (*p* < 0.004, all tests). Furthermore, dMCI and aMCI participants obtained a higher MMSE score than dementia participants (*p* < 0.001, both analyses; Table 3).

**Table 2:** *K*-mean cluster analysis (*z*-scores).

	**Normal (*n* = 23)**	**Amnestic MCI (*n* = 17)**	**Dysexecutive MCI (*n* = 23)**	**Dementia (*n* = 14)**
P(r)VLT—delayed free recall	1.011	−0.976	0.395	−1.125
P(r)VLT—recognition hits	0.648	−0.614	0.675	−1.428
BDST—SERIAL order recall	0.923	0.322	−0.348	−1.334
“Animal” fluency (total responses)	1.026	−0.191	−0.282	−0.990

P(r)VLT, Philadelphia (repeatable) Verbal Learning Test; BDST, Backward Digit Span Test.

**Table 3:** Demographic and clinical information: means and standard deviations.

	**Normal (*n* = 23)**	**Amnestic MCI (*n* = 17)**	**Dysexecutive MCI (*n* = 23)**	**Dementia (*n* = 14)**	**Significance**
Age	70.04 (8.74)	74.82 (7.11)	75.00 (9.66)	79.93 (7.04)	dem > NC; *p* < 0.004
Education	16.30 (3.02)	14.88 (3.46)	14.39 (2.79)	12.36 (2.81)	dem < NC; *p* < 0.001
MMSE	29.50 (0.79)	25.88 (2.42)	27.83 (1.72)	20.09 (4.18)	dem < all groups; *p* < 0.001
					aMCI < NC; *p* < 0.004
Executive index score (EIS; *z*-score)	0.97 (0.38)	0.11 (0.52)	−0.31 (0.39)	−1.13 (0.42)	dem < all groups; *p* < 0.001
					dMCI < aMCI & NC; *p* < 0.030
Memory index score (MIS; *z*-score)	0.83 (0.22)	−0.80 (0.43)	0.53 (0.30)	−1.23 (0.50)	dem < dMCI & NC; *p* < 0.001
					aMCI < dMCI & NC; *p* < 0.001
					aMCI = dem; ns
Present female	25.6%	18.6%	39.5%	16.3%	

aMCI, amnestic mild cognitive impairment; dMCI, dysexecutive mild cognitive impairment.

### Comparing cluster analysis classification vs. clinical diagnosis and correlations between paper and pencil neuropsychological tests

As described above, 47 of the research participants underwent a comprehensive neuropsychological assessment. Diagnosis derived from comprehensive neuropsychological assessment vs. cluster-determined classification found 100% agreement for patients believed to be presenting with dementia (*n* = 10). The agreement regarding cognitively unimpaired (*n* = 15) was 80%, where cluster-determined classification characterized one participant with aMCI and two participants with dMCI. Comprehensive assessment characterized 22 patients with subtle cognitive impairment (SCI; *n* = 14) and aMCI (*n* = 8). When all of these patients were aggregated into a single SCI/MCI group, there was 90.90% agreement with the cluster-determined classification, where two patients were statistically classified into the dementia group. Across all cluster-determined groups, the digitally administered neuropsychological tests successfully classified 93.33% of participants (sensitivity = 1, specificity = 0.8, positive predictive value = 0.88, and negative predictive value = 1).

As displayed in Supplementary Table 1, there were robust correlations between BDST percent ANY and SERIAL recall and their analogous paper and pencil executive tests that assessed auditory span and verbal/visual working memory. Similar correlations were also obtained between the P(r)VLT Delayed Free Recall and the P(r)VLT Recognition False Positive/Correct Hit Ratio, as well as between the CVLT-II Delay Free Recall and CVLT Recognition Discriminability Index (Rascovsky et al., 2007). Moreover, significant correlations were also found between performance on the Wechsler Adult Intelligence Scale-III (Wechsler, 1997) Similarities subtest, total “animal” fluency output, and “animal” semantic association.

#### P(r)VLT within-group contrast comparisons: retention and forgetting

For aMCI participants, P(r)VLT delayed free recall was lower than immediate free recall, trial 2 [*t*_(16)_ = 3.17; *p* < 0.006]. Other within-group contrast comparisons were not significant. An opposite profile emerged for the dMCI group, where a better score for P(r)VLT recognition hits was observed in relation to immediate free recall trial 2 [*t*_(22)_= 2.97, *p* < 0.007[ and delayed free recall [*t*_(22)_ = 2.15, *p* < 0.042]. For the CU group, there were marginal differences when P(r)VLT immediate free recall, trial 2, was compared to recognition hits [*t*_(22)_ = 3.02; *p* < 0.006] and when delay-free recall was compared to recognition hits [*t*_(22)_ = 3.89; *p* < 0.001].

#### P(r)VLT free recall semantic cluster responses, extra-list intrusion errors, perseverations, and total recognition foils

The MANOVA for P(r)VLT semantic cluster responses, extra-list intrusion errors, perseverations, and recognition foils were significant [*F*_(12, 200)_ = 14.14, *p* < 0.001, η^2^ = 0.459]. CU participants produced more semantic cluster responses than aMCI participants (*p* < 0.002). Dementia participants generated more extra-list intrusion errors than CU and aMCI participants (*p* < 0.005, both tests). No differences were obtained for perseverations. aMCI participants erroneously endorsed more total recognition foils than CU and dMCI participants (*p* < 0.001, both tests). Dementia participants also erroneously endorsed more total recognition foils than all groups (*p* < 0.006, all tests; Table 4).

**Table 4:** Digital neuropsychological protocol: error, process and related outcome measures (means and standard deviations).

	**Normal (*n* = 23)**	**Amnestic MCI (*n* = 17)**	**Dysexecutive MCI (*n* = 23)**	**Dementia (*n* = 14)**	**Significance**
**Philadelphia (repeatable) Verbal Learning Test (PrVLT)**					
P(r)VLT: free recall semantic cluster responses	2.52 (2.44)	0.29 (0.58)	1.39 (1.69)	0.66 (1.04)	NC > aMCI; *p* < 0.002
P(r)VLT: free recall extra-list intrusion errors	0.13 (0.45)	0.58 (1.00)	0.04 (0.20)	1.33 (1.67)	dem > aMCI; *p* < 0.002
					dem > dMCI. *p* < 0.005
P(r)VLT: free recall perseverations	0.34 (0.64)	0.05 (0.24)	0.08 (0.28)	0.00 (0.00)	ns
P(r)VLT: total recognition foils	0.08 (0.28)	2.05 (1.29)	0.00 (0.00)	3.13 (1.30)	dem > all groups; *p* < 0.006
					aMCI > dMCI & NC; *p* < 0.001
P(r)VLT: delay free recall/recognition foil ratio	0.98 (0.04)	0.65 (0.21)	1.00 (0.00)	0.46 (0.24)	NC > aMCI & dem; *p* < 0.001
					dMCI > aMCI & dem; *p* < 0.001
					aMCI > dem; *p* < 0.002
Recognition latency (reaction time; msecs)	5,985.67 (858.01)	7,673.00 (2,039.87)	6,818.74 (790.35)	8,566.75 (3,026.09)	NC < aMCI & dem; *p* < 0.009
					dMCI < dem; *p* < 0.006
**“Animal” Semantic Fluency Test**					
“Animal” Association Index	3.18 (0.42)	2.96 (0.39)	3.16 (0.62)	2.61 (0.68)	dMCI > dem; *p* < 0.023
					NC > dem; *p* < 0.060
**Backward Digit Span Test**					
BDST total out-of-sequence transposition errors	1.52 (1.95)	2.76 (2.04)	5.60 (2.49)	7.33 (2.74)	dem > aMCI & NC; *p* < 0.001
					dMCI > aMCI & NC; *p* < 0.001
BDST total dysexecutive errors	0.47 (0.73)	1.70 (1.21)	1.34 (0.93)	2.73 (1.22)	dem > all groups; *p* < 0.049
					dMCI > NC; *p* < 0.050

P(r)VLT, Philadelphia (repeatable) Verbal Learning Test; aMCI, amnestic mild cognitive impairment; dMCI, dysexecutive mild cognitive impairment; ns, not significant.

#### P(r)VLT prototypic vs. generic recognition foils

The MANOVA for P(r)VLT prototypic and generic recognition foils was significant [*F*_(6, 138)_ = 26.56, *p* < 0.001, η^2^ = 0.536]. Dementia participants endorsed more generic recognition foils than dMCI participants (*p* < 0.008), and aMCI endorsed more generic foils than dMCI and CU participants (*p* < 0.001). Moreover, dementia participants endorsed more prototypic foils than all other groups (*p* < 0.001, all tests). Similarly, aMCI participants endorsed more prototypic foils than dMCI and CU participants (*p* < 0.001).

#### P(r)VLT recognition test performance

The group effect for recognition latency for correct recognition test trials was significant [*F*_(3, 74)_ = 7.70, *p* < 0.001, η^2^ = 0.254]. Faster recognition latencies were observed for CU compared to aMCI and dementia groups (*p* < 0.009, both tests) and dMCI participants compared to dementia participants (*p* < 0.006). The effect for the group on the recognition hit/recognition false positive ratio was also significant [*F*_(3, 70)_ = 50.21, *p* < 0.001, and η^2^ = 0.683]. Both CU and dMCI participants obtained higher ratios than the aMCI and dem groups. Furthermore, aMCI participants obtained a higher ratio compared to dem participants (*p*<*p* < 0.002).

A regression analysis was performed to assess relations between recognition latency for correct test items and P(r)VLT recognition foil subtypes. For this analysis, age, education, and sex were entered into Block 1, followed by prototypic and generic foils entered into Block 2. Slower recognition latency was found in relation to the production of greater numbers of prototypic recognition foils (*R*^2^ = 0.308, df = 2, 70, *F* = 11.80, *p* < 0.001; prototypic foils, beta = 0.497, *p* < 0.001). A similar regression analysis was significant, examining relations between latencies for correct recognition responses and P(r)VLT total free recall semantic cluster responses and total free recall extra-list intrusion errors (*R*^2^ = 0.160, df = 2, 70, *F* = 3.57, *p* < 0.033). Slower latency was found in relation to the production of greater numbers of free recall extra-list intrusion errors (intrusion errors, beta= 0.225, *p* < 0.050).

### Backward Digit Span Test transposition and dysexecutive errors

The MANOVA for transposition and dysexecutive errors was significant [*F*_(6, 138)_ = 12.67, *p* < 0.001, η^2^ = 0.355). Both dementia and dMCI groups made more transposition errors than aMCI and CU groups (*p* < 0.001, all analyses); similarly, dementia and dMCI groups made more dysexecutive errors than aMCI and CU groups (*p* < 0.050, all analyses). aMCI participants also made more dysexecutive errors than CU participants (*p* < 0.003).

### “Animal” Fluency Association Index

The group effect for “animal” AI was significant [*F*_(3, 76)_ = 3.38, *p* < 0.023, η^2^ = 0.127], where dMCI participants produced a higher AI compared to dementia participants (*p* < 0.023). A regression analysis (*R*^2^ = 0.184, df = 2, 72, *F* = 5.24, *p* < 0.008) found a lower “animal” AI was associated with the production of a greater number of prototypic recognition foils (beta = −0.284, *p* < 0.014). A regression analysis examining the association between the “animal” AI and P(r)VLT semantic cluster responses and the emergence of extra-list intrusion errors was not significant.

### Digital core summary scores

The MANOVA that assessed for between-group differences for the digital MIS and EIS index scores was significant [*F*_(6, 136)_ = 81.88, *p* < 0.001, η^2^ = 0.783; Table 3]. Subsequent analyses found that on the MIS, almost all groups were dissociated from each other (*p* < 0.001, all analyses), except for equal performance when dMCI and CU participants were compared and equally impaired performance between aMCI and dementia participants. On the EIS, all groups were differentiated from each other (*p* < 0.030).

Within-group, aMCI participants scored lower on the memory compared to the executive index [*t*_(15)_ = 4.57, *p* < 0.001]. An opposite profile was obtained for dMCI participants, where there was a lower score on the executive vs. the memory index [*t*_(22)_ = 8.13, *p* < 0.001]. Similar within-group analyses for dementia and CU participants were not significant.

## Discussion

The current research was undertaken to assess how well a brief, 10-min, digitally administered and scored neuropsychological protocol could classify participants into their respective groups, compare these classifications against comprehensive neuropsychological assessment, and assess how well this technology can measure and tally errors and the processes employed to bring tests to fruition. The overall strategy used in the construction of the digital neuropsychological protocol was to employ assessment paradigms that are well-known, thoroughly researched, and have been shown to differentiate between patient groups with different forms of dementia and MCI (for reviews, see Lamar et al., 2010; Libon et al., 2018). An additional overarching goal was to develop a digitally administered/scored protocol that can leverage mobile technologies to generate features that characterize patient performance with similar sensitivity and precision as would be encountered by an expert neuropsychologist. In order to ensure some continuity between traditional paper and pencil tests and new, emerging digital assessment technology, the digitally administered/scored protocol was designed to calculate a panel of core, well-understood outcome metrics derived from paper and pencil tests, along with digitally derived scores that quantify patterns of errors and process-based strategies.

The cluster solution described above, using four core variables, classified research participants into four well-understood groups representing intact test performance: dementia, aMCI, and dMCI. Moreover, the analyses described above are consistent with decades of research that have operationally defined the presence and severity of amnesia (see Butters and Miliotis, 1985; Bauer et al., 2012 for review), as well as dysexecutive difficulty commonly seen in AD and MCI (Libon et al., 2011, 2018; Eppig et al., 2012). Further, the robust correlations obtained between digitally administered/scored assessments of verbal episodic memory and executive control and their analogous paper and pencil measures suggest good concurrent validity.

Among the aMCI group, immediate free recall on a serial list learning test was generally intact. However, an amnestic state is defined, in part, by a precipitous decline in recall after a delay, with no improvement when patients are assessed with a recognition test condition (Butters and Miliotis, 1985). In this context, aMCI participants exhibited a striking decline from immediate free recall to delayed free recall. Moreover, there was an equal level of impairment when delayed free recall was compared to the delayed recognition test condition—a pattern of behavior seen in previous research where dementia and MCI subtypes were studied (Price et al., 2009; Libon et al., 2011). By contrast, dMCI participants demonstrated improvement when delayed recognition was compared to delayed free recall test performance. This pattern of performance suggests a retrieval, rather than an encoding problem, and has been shown to typify dementia and MCI subtypes where dysexecutive, rather than amnestic impairment, is the most prominent feature (Price et al., 2009; Libon et al., 2011; Eppig et al., 2012).

Past research also suggests that an amnestic state often presents with many free recall extra-list intrusion errors and profligate responses to delayed recognition foils (Delis et al., 1991; Bondi et al., 1994; Price et al., 2009; Thomas et al., 2018). In the current research, statistical differences were not found for the production of free recall extra-list intrusion errors; however, profligately responding to delayed recognition foils was a striking feature that distinguished both cluster-determined aMCI and dementia patients as compared to other groups.

To the best of our knowledge, latency in responding to correct, verbal serial list learning recognition test items has never been studied. As noted above, recognition latency was faster for CU and dMCI participants compared to aMCI and dementia participants. This is perhaps not surprising given the absence of any indication of clinical amnesia among CU and dMCI participants. Moreover, slower recognition latency for correct recognition trials was observed in relation to the production of highly prototypic semantic foils, suggesting that there could be a specific relationship between this new process variable and degraded semantic stores (Libon et al., 2013). Additional research is necessary to explore this possibility. Future research should investigate whether latency or reaction time on P(r)VLT-free recall test trials might also differentiate between groups. Overall, the digital protocol appears to be able to define many of the neurocognitive constructs and underlying processes associated with amnesia when assessed using a verbal serial list learning test.

Dysexecutive impairment is commonly observed in patients with mild AD and MCI (Eppig et al., 2012; Libon et al., 2018) and was assessed in the current research with the Backward Digit Span Test (BDST). This test was inspired by Kaplan et al. (1991), who compiled a nosology of error subtypes and suggested that there was added value in scoring whether each digit in the test trial was either correct or incorrect. Using these strategies, prior research has found marked impairment for serial order recall among vascular dementia (VaD) patients compared to AD patients (Lamar et al., 2007, 2008) and memory clinic patients classified statistically with dysexecutive MCI (Eppig et al., 2012).

Emrani et al. (2018) also studied the production of BDST errors among memory clinic patients with MCI subtypes. These researchers highlighted the emergence of a greater number of both items as well as out-of-sequence transposition errors among their mixed/dysexecutive patients. Indeed, the behavior regarding the production of BDST errors described by these authors is highly similar to that obtained in the current research. Interestingly, there were no differences between the backward digit and SERIAL order recalls when CU and aMCI participants were compared. Intact backward digit SERIAL order recall in the context of striking evidence for memory impairment in the aMCI group provides additional evidence for the capacity of the digital protocol to operationally define important neurocognitive constructs and phenotypes associated with neurodegeneration.

The semantic (“animal”) fluency test provides an assessment of both executive and language-related behavior in the context of a lexical search. Imaging research with CU participants suggests that both “animal” and letter (“FAS”) fluency tests are associated with a wide network of brain regions involving anterior cingulate, left prefrontal, and left temporal brain regions, with greater temporal activation associated with “animal” fluency test performance (Mummery et al., 1996; Gourovitch et al., 2000; Libon et al., 2009).

The “animal” AI was constructed to measure the strength of the semantic network's putatively underlying aggregate output. In the current research, a better “animal” Association Index score was found for CU and dMCI compared to dementia participants. Equally interestingly, regression analysis found that lower “animal” AI scores were associated with the production of a greater number of P(r)VLT prototypic recognition foils. Further research is necessary to explore how output on animal fluency and its underlying implications for semantic networks can be leveraged to differentiate between patient groups. Finally, the digital protocol yields two summary scores measuring episodic memory and executive control. As described above, both between- and within-group analyses of both index scores were able to differentiate between cluster-determined groups, suggesting that metrics that summarize performance on the digitally administered/scored protocol by aggregating several features also contain sufficient information to dissociate cognitive phenotypes.

The Mini-Mental State Examination (Folstein et al., 1975) and the Montreal Cognitive Assessment (MoCA; Nasreddine et al., 2005) are commonly used to screen for dementia and MCI syndromes. This digital neuropsychological protocol requires less time than either of these tests. The advantages of this protocol over the MMSE and MoCA include a panel of commonly used core analog scores, an additional panel of scores that measure errors and processes, and separate summary scores measuring episodic memory and executive control, the two most commonly impaired neurocognitive constructs associated with neurodegenerative illnesses.

The current research is not without limitations. First, only a modest sample was available for these analyses. Additional research using a larger sample is needed to verify the statistical relationships described above. Second, research using additional types of neurodegenerative illness would expand upon the statistical findings reported above and the replicability of the findings. Finally, as observed in prior research (Giannouli, 2023), there can be substantial variability regarding older people's familiarity with and attitude toward AI technology. This factor needs to be considered in future research.

Nonetheless, the current research has several strengths. First all subtests are based on clinical assessment and research paradigms that have been thoroughly researched. Second, digital administration and scoring maximize reliability for test administration and the absence of any subjectivity in scoring and error tabulation. Third, core variables were highly accurate in classifying CU, MCI, and dementia groups, as confirmed by group differences for novel process metrics. Finally, the strong correlations between digitally obtained measures assessing verbal episodic memory and executive control and their paper and pencil analog measures suggest reasonable concurrent validity.

In summary, the results of the current research suggest that, when brought to scale and deployed in longitudinal research or clinical environments, this digital neuropsychological protocol could uncover subtle, highly nuanced behavior that might predict the emergence of ADRD and MCI syndromes. In the context of the development of pharmacologic therapies that hold promise to treat neurodegenerative illnesses, a digital protocol leveraging a digital platform able to analyze process-based metrics could be a powerful tool to screen and identify patients who might benefit from these therapies.

## Data availability statement

The raw data supporting the conclusions of this article will be made available by the authors, without undue reservation.

## Ethics statement

The studies involving humans were approved by Institutional Review Board, Rowan University. The studies were conducted in accordance with the local legislation and institutional requirements. The Ethics Committee/Institutional Review Board waived the requirement of written informed consent for participation from the participants or the participants' legal guardians/next of kin because Data obtained as part of routine clinical assessment.

## Author contributions

DL: Writing – review & editing, Writing – original draft, Formal analysis, Data curation, Conceptualization. RS: Writing – review & editing, Writing – original draft, Conceptualization. CP: Writing – review & editing, Writing – original draft, Conceptualization. ML: Writing – review & editing, Writing – original draft, Conceptualization. SC: Writing – review & editing, Writing – original draft, Conceptualization. OB: Conceptualization, Writing – review & editing. MK: Writing – review & editing. ST: Conceptualization, Writing – review & editing, Writing – original draft. AJ: Writing – review & editing. RB: Writing – review & editing. AP-L: Writing – review & editing.
