# Supplementary material for: Digital assessment of cognition in neurodegenerative disease: a data driven approach leveraging artificial intelligence
Source: Front Psychol. 2024 Jul 5;15:1415629. doi: 10.3389/fpsyg.2024.1415629 (PMC11258860; doi:10.3389/fpsyg.2024.1415629)
Supplement: Supplementary file 1 [file Table_1.DOCX]

| **Supplemental Table 1: Correlation Between Core Digital Neuropsychological Outcome Variables and Paper/ Pencil Neuropsychological Test** | | |
| --- | --- | --- |
|  | **BDST Percent ANY order recall** | **BDST Percent SERIAL Recall** |
| **KBNA Sequence**  **Subtest** | 0.667; p< 0.001 | 0.707; p< 0.001 |
| **WAIS-IV Digits**  **Forward** | 0.399; p< 0.010 | 0.459; p< 0.003 |
| **WAIS-IV Digits**  **Backward** | 0.357, p< 0.022 | 0.589; p< 0.001 |
| **WMS-IV Symbol Span**  **Subtest** | 0.492; p< 0.002 | 0.474; p< 0.003 |
|  | **P(r)VLT Delayed Free Recall** | **P(r)VLT Delay Free Recall/ Recognition Foil Ratio** |
| **CVLT Long Delay Free**  **Recall** | 0.682; p< 0.001 | 0.653; p< 0.001 |
| **CVLT Recognition**  **Discriminability** | 0.613; p< 0.001 | 0.765; p< 0.001 |
|  | **Semantic (‘animal’) Fluency Total Responses** | **‘animal’ Association**  **Index** |
| **WAIS-III Similarities**  **Subtest** | 0.460; p< 0.002 | 0.394; p< 0.011 |
| **Boston Naming**  **Test** | 0.274; ns | -0.171; ns |
| KBNA= Kaplan Baycrest Neuropsychological Assessment; WAIS= Wechsler Adult Intelligence Scale; WMS= Wechsler Memory Scale; CVLT= California Verbal Learning Test; ns= not significant | | |

| **Supplemental Table 2. Digital Neuropsychological Protocol Core Output Measures:**  **(Means & Standard Deviations)** | | | | | |
| --- | --- | --- | --- | --- | --- |
|  | **normal**  **(n= 23)** | **amnestic MCI (n= 17)** | **dysexecutive MCI (n= 23)** | **dementia**  **(n= 14)** | p |
| **Philadelphia (repeatable) Verbal Learning Test (PrVLT)** | | | | | |
| P(r)VLT: immediate free recall;  trial 1 (range 0-6) | 4.69  (0.63) | 3.05  (1.02) | 3.60  (0.89) | 1.60  (1.18) | NC > all groups; p< 0.001  dem < aMCI & dMCI; p< 0.001 |
| P(r)VLT: immediate free recall; trial 2 (range 0-6) | 5.56  (0.50) | 3.88  (1.05) | 4.65  (1.02) | 2.80  (0.86) | NC > aMCI & dem; p< 0.001  dMCI > aMCI & dem; p< 0.001 |
| P(r)VLT: delayed free recall;  (range 0-6) | 4.73  (0.91) | 0.52  (0.71) | 3.43  (1.27) | 0.20  (0.56) | NC > all groups; p< 0.001  dMCI > aMCI & dem; p< 0.001 |
| P(r)VLT: recognition  hits (range 0-6) | 5.95  (0.20) | 3.94  (1.29) | 5.90  (0.23) | 2.80  (1.47) | NC > aMCI & dem; p< 0.001  dMCI > aMCI & dem; p< 0.001  aMCI > dem; p< 0.003 |
| **‘animal’ Semantic Fluency Test** | | | | | |
| Total Responses  (60 seconds) | 21.39  (4.67) | 14.17  (4.74) | 13.26  (3.91) | 8.93  (3.61) | NC > all groups; p< 0.001  dem < aMCI & dMCI; p< 0.040 |
| **Backward Digit Span Test** | | | | | |
| ANY order  Recall (percent) | 98.26  (3.60) | 93.12  (6.50) | 93.76  (5.88) | 79.10  (13.50) | dem < all groups; p< 0.001 |
| SERIAL order  Recall (percent) | 88.69  (14.27) | 72.93  (17.23) | 55.34  (17.80) | 30.66  (13.52) | NC > all groups; p< 0.050  dMCI < aMCI & dem; p< 0.005 |
| P(r)VLT= Philadelphia (repeatable) Verbal Learning Test; aMCI= amnestic mild cognitive impairment  dMCI= dysexecutive mild cognitive impairment; ns= not significant | | | | | |
